# A survey of parental experiences while viewing MRI images at a fetal care center

**DOI:** 10.1038/s41372-025-02319-9

**Published:** 2025-05-17

**Authors:** Nicholas D. O’Connor, Louis J. Martin, Rachel L. Tullar, Brandon P. Brown, Zeynep N. I. Salih

**Affiliations:** 1 Indiana University, School of Medicine, Department of Medicine, Indianapolis, IN USA; 2 Indiana University, School of Medicine, Department of Pediatrics, Indianapolis, IN USA; 3 Indiana University, School of Medicine, Department of Radiology & Imaging Sciences, Indianapolis, IN USA

**Keywords:** Health care, Medical ethics

## Introduction

Fetal MRI (fMRI) is now recognized as the standard imaging when abnormalities are detected on the routine US [1]. Prior research focusing on decreasing anxiety during the MRI screening, found that parental anxiety also largely stemmed from anticipation of how the MRI results would be interpreted [2, 3]. Although it was shown that the fMRI images were found valuable by the expectant couples [4] there is a paucity of data exploring parental experiences while viewing their baby’s MRI images in an interdisciplinary fetal care conference setting, the way increasingly used to counsel parents at fetal centers [5]. In this study, we aimed to explore parental experiences while viewing fMRI images as part of fetal care conferences, specifically how viewing MRI images has affected parents’ emotions, feelings of attachment to their baby, and decision-making for their baby.

## Methods

A web-based survey was created using a literature review, expert opinion, and parent input (Supplement 1). Parental responses were asked using a Likert scale (1–5 scoring). Inclusion criteria were 18 years of age or older, English-speaking, and having the fMRI images reviewed at a care conference with a group of specialists, including the pediatric radiologist at a Midwest Maternal-Fetal Center. Each woman received the survey via email 3 days after her care conference, with a single follow-up reminder sent 5 days after the initial survey invitation over a 6-month period in 2023. To understand whether emotional responses to the baby’s MRI differed as a function of the mother’s age, race, ethnicity, or education, correlations were performed using Kendall’s tau-b (τb). For each set of correlations, *p* values were adjusted using the Holm-Bonferroni method.

Free text responses were qualitatively analyzed using thematic analysis [6].

## Results

**Fig. 1:**
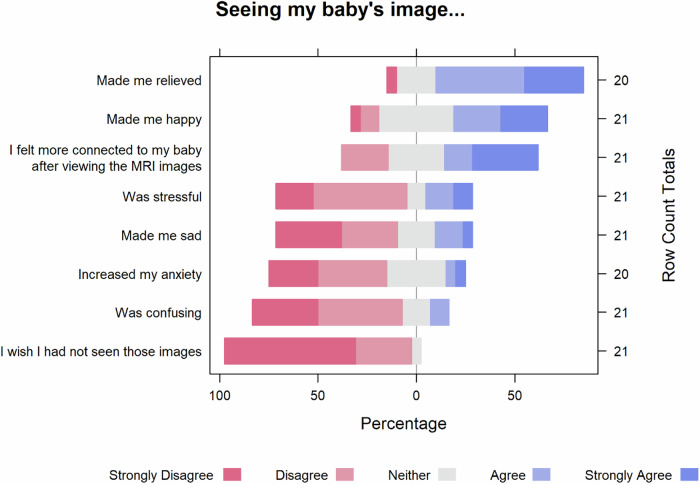
Likert plots showing how much participants agreed or disagreed with statements about their emotions after seeing MRI images at the fetal care conference.

74 mothers received the survey, 21 responded (28% response rate). Participants were 18–44 years old, and all had completed at least high school (Supplement. 2). Overall, most parents agreed that viewing their baby’s images with the radiologist made them feel relieved, happy, and more connected to the baby (Fig. 1). While most mothers did not report that viewing the MRI images had affected their attachment to the baby, responses to decision-making varied (Supplement 3). No parent, even a subset of parents whose babies carried a lethal diagnosis, wished not to see the MRI images of their baby. Older age was associated with less relief/happiness and more anxiety/confusion upon seeing the baby’s images (each *p* < 0.05, Kendall’s tau-b). These correlations were no longer significant after correcting for multiple comparisons, likely because of the small sample size. No correlation was observed between emotional responses to viewing the MRI images with maternal age, educational level, race, or ethnicity. Qualitative analysis of 14 free text responses yielded three themes (Supplement 4): 1. Viewing the images provides clarity. 2. The expert explanation is impactful. 3. Appreciating having a choice for viewing the images.

## Discussion

This is the first study exploring parental experiences viewing fMRI images with the radiologist at a fetal interdisciplinary care conference. As shown by others [7], radiologists’ communication skills at the care conference and also hearing the interpretations of other specialists might have helped women navigate the information provided by the fMRI images, made them feel relieved, and actually made most mothers feel more connected to their baby [3] Several parents commented on how seeing what was going on with their baby provided clarity and helped them make a plan for the baby. This is consistent with other research showing the fMRI imaging being helpful in experiences of uncertainty [4] Although most parents do not find viewing the images to be a stressful, confusing, or anxiety-increasing event, nor do they express any regret, older parents may be experiencing less relief/happiness and more anxiety/confusion upon seeing their baby’s images, thus requiring a different approach to their emotive needs. Viewing the MRI images had varying effects on parental decision-making, which had not been reported in previous research. It requires further studies to understand how viewing the MRI images in an interdisciplinary care setting affects parental decision-making.

Our study’s main limitations are the low response rate and the inclusion of only English-speaking parents, which might have led to bias in the responses. Further research, using qualitative interview methods, should include a diverse group of participants and fathers to explore their met and unmet needs, especially emotive and decision-making needs, while viewing fMRI images in a fetal care conference setting and how clinicians could improve their counseling accordingly.

## Supplementary information

Supplement 1

Supplement 2

Supplement 3

Supplement 4
