# Supplementary material for: A survey of parental experiences while viewing MRI images at a fetal care center
Source: J Perinatol. 2025 May 17;45(9):1300–1. doi: 10.1038/s41372-025-02319-9 (PMC12431840; doi:10.1038/s41372-025-02319-9)
Supplement: Supplementary file 1 — Supplement 1 [file 41372_2025_2319_MOESM1_ESM.docx]

Supplement 1-Survey Questions

| How satisfied were you with the time spent reviewing your baby's MRI during the care conference? | Very Satisfied  Satisfied  Unsure  Dissatisfied  Very Dissatisfied |
| --- | --- |
| Seeing the MRI images clarified my baby's condition. | Strongly Disagree  Disagree  Neither agree nor disagree  Agree  Strongly Agree |
| The radiologist helped me better understand the MRI findings. | Strongly Disagree  Disagree  Neither agree nor disagree  Agree  Strongly Agree |
| Please explain why seeing the MRI images did not give you clarity | (Free Text) |
| Was the radiologist was present in person during the care conference? | Yes  No  I do not know who the radiologist was |
| Did you find it helpful that the radiologist pointed to the images in person? | Yes  No |
| Would it have been helpful if the radiologist had been in the care conference and shown images in person? | Yes  No |
| I was able to ask questions about the MRI findings that were important to me. | Strongly Disagree  Disagree  Neither agree nor disagree  Agree  Strongly Agree |
| I would like more written information about the MRI results to take with me. | Strongly Disagree  Disagree  Neither agree nor disagree  Agree  Strongly Agree |
| Stressful | Strongly Disagree  Disagree  Neither agree nor disagree  Agree  Strongly Agree |
| Confusing | Strongly Disagree  Disagree  Neither agree nor disagree  Agree  Strongly Agree |
| Made me sad | Strongly Disagree  Disagree  Neither agree nor disagree  Agree  Strongly Agree |
| Made me happy | Strongly Disagree  Disagree  Neither agree nor disagree  Agree  Strongly Agree |
| Made me relieved | Strongly Disagree  Disagree  Neither agree nor disagree  Agree  Strongly Agree |
| Increased my anxiety | Strongly Disagree  Disagree  Neither agree nor disagree  Agree  Strongly Agree |
| I wish I had not seen those images | Strongly Disagree  Disagree  Neither agree nor disagree  Agree  Strongly Agree |
| Please elaborate why the images made you feel this way | (Free Text |
| I felt more connected to my baby after viewing the MRI images | Strongly Disagree  Disagree  Neither agree nor disagree  Agree  Strongly Agree |
| Seeing my baby's MRI images affected my decision-making for the care of my baby | Strongly Disagree  Disagree  Neither agree nor disagree  Agree  Strongly Agree |
| How likely are you to recommend viewing MRI images of their baby to other parents? | Extremely likely  Likely  Neutral  Unlikely  Extremely unlikely |
| Is there anything you want to share about viewing your baby's MRI images that day? | (Free Text) |
| What is your relationship to the baby | Mother  Father |
| Who was with you at the care conference where you viewed the MRI images? | Spouse / Partner  A Family Member  A friend  Other (Free Text)  I was alone |
| What is your age? | 18-24  25-34  35-44 |
| What is the highest level of education you have completed? | Grade School  Junior High  High School or GED  Associate Degree  Bachelor's Degree  Master's Degree  Ph.D., MD, JD, or other terminal degrees  N/A |
| Race | African American or Black  Asian  Caucasian or White  American Indian and/or Alaska Native  Native Hawaiian or Pacific Islander  Other  Prefer not to answer |
| Ethnicity | Hispanic and/or Latino  Not Hispanic and/or Latino |
