# Supplementary material for: A survey of parental experiences while viewing MRI images at a fetal care center
Source: J Perinatol. 2025 May 17;45(9):1300–1. doi: 10.1038/s41372-025-02319-9 (PMC12431840; doi:10.1038/s41372-025-02319-9)
Supplement: Supplementary file 2 — Supplement 2 [file 41372_2025_2319_MOESM2_ESM.docx]

Supplement 2-Demographics of participants

| *Demographics (n = 19)* | Count (%) |
| --- | --- |
| **Age** |  |
| 18-24 | 3 (15.8) |
| 25-34 | 10 (52.6) |
| 35-44 | 6 (31.6) |
| **Education Level** |  |
| High School or GED | 7 (36.8) |
| Associate Degree | 1 (5.3) |
| Bachelor’s Degree | 6 (31.6) |
| Master’s Degree | 5 (26.3) |
| **Race** |  |
| Black | 2 (9.1) |
| Asian | 1 (4.5) |
| Caucasian or White | 13 (59.1) |
| Other | 3 (13.6) |
| **Ethnicity** |  |
| Hispanic or Latino | 2 (10.5) |
| Not Hispanic or Latino | 17 (89.5) |
| **Relationship to Baby** |  |
| Mother | 19 (100.0) |
| **Who accompanied you?** |  |
| Spouse/Partner | 14 (63.6) |
| Family Member | 2 (9.1) |
| Friend | 1 (4.5) |
| Alone | 2 (9.1) |
