# Supplementary material for: A survey of parental experiences while viewing MRI images at a fetal care center
Source: J Perinatol. 2025 May 17;45(9):1300–1. doi: 10.1038/s41372-025-02319-9 (PMC12431840; doi:10.1038/s41372-025-02319-9)
Supplement: Supplementary file 3 — Supplement 3 [file 41372_2025_2319_MOESM3_ESM.docx]

Supplement 3 -Responses to survey questions

| Question | Strongly Agree  N (%) | Agree | Neither | Disagree | Strongly Disagree  N (%) |
| --- | --- | --- | --- | --- | --- |
| Seeing the MRI images clarified my baby's condition. | 12 (63.2%) | 2 (10.5%) | 3 (15.8%) | 0 (0%) | 2 (10.5%) |
| Seeing my baby's MRI images affected my decision-making for the care of my baby | 3 (15.8%) | 3 (15.8%) | 6 (31.6%) | 3 (15.8%) | 4 (21.1%) |
| I was able to ask questions about the MRI findings that were important to me. | 14 (73.7%) | 4 (21.1%) | 1 (5.3%) | 0 (0%) | 0 (0%) |
| I would like more written information about the MRI results to take with me. | 3 (15.8%) | 6 (31.6%) | 8 (42.1%) | 2 (10.5%) | 0 (0%) |
| Seeing the MRI images was  Stressful | 2 (10.5%) | 3 (15.8%) | 0 (0%) | 10 (52.6%) | 4 (21.1%) |
| Confusing | 0 (0%) | 1 (5.3%) | 2 (10.5%) | 9 (47.4%) | 7 (36.8%) |
| Made me sad | 1 (5.3%) | 3 (15.8%) | 2 (10.5%) | 6 (31.6%) | 7 (36.8%) |
| Made me happy | 5 (26.3%) | 5 (26.3%) | 6 (31.6%) | 2 (10.5%) | 1 (5.3%) |
| Made me sad | 1 (5.3%) | 3 (15.8%) | 2 (10.5%) | 6 (31.6%) | 7 (36.8%) |
| Increased my anxiety | 1 (5.6%) | 1 (5.6%) | 4 (22.2%) | 7 (38.9%) | 5 (27.8%) |
| I wish I had not seen those images | 0 (0%) | 0 (0%) | 1 (5.3%) | 5 (26.3%) | 13 (68.4%) |
| I felt more connected to my baby after viewing the MRI images | 7 (36.8%) | 3 (15.8%) | 5 (26.3%) | 4 (21.1%) | 0 (0%) |
|  |  |  |  |  |  |
