# Supplementary material for: A survey of parental experiences while viewing MRI images at a fetal care center
Source: J Perinatol. 2025 May 17;45(9):1300–1. doi: 10.1038/s41372-025-02319-9 (PMC12431840; doi:10.1038/s41372-025-02319-9)
Supplement: Supplementary file 4 — Supplement 4 [file 41372_2025_2319_MOESM4_ESM.docx]

Supplement 4: Qualitative Analysis of Free Text Responses

| **Themes** | **Quotes** |
| --- | --- |
| **Viewing the images provides clarity** | *“I was glad I could see what was going on with my baby even if it made me sad. It showed me a clear plan for my baby and new discoveries”*  *“The images made me sad, just because the chances of his survival are not high, but I was also relieved to see the concerns with my own eyes and leave with answers”* |
| **The expert explanation is impactful** | *“It didn’t increase my anxiety seeing them because the radiologist was able to explain what I was looking at”*  *“loved the thorough explanation about each mri image and slicing”* |
| **Appreciating having a choice for viewing the images** | *“I just really appreciated the choice. The doc viewing the images asked if we wanted to go over them. If we had bad news I may not have wanted to see them so the choice was nice”* |
